# Enhancing Patient-Centered Health Technology Assessment: A Modified Delphi Panel for PICOS Scoping in Spinal Muscular Atrophy

**DOI:** 10.3390/jmahp14010006

**Published:** 2026-01-19

**Authors:** Emanuele Arcà, Adele Barlassina, Adaeze Eze, Valentina Strammiello

**Affiliations:** 1 Strategic Market Access, OPEN Health, 3068 AV Rotterdam, The Netherlands; 2 Patient-Centered Outcomes, OPEN Health, 3068 AV Rotterdam, The Netherlands; 3 European Patients’ Forum, 1040 Brussels, Belgium

**Keywords:** health technology assessment, PICOS scoping, Delphi consensus, patient engagement, stakeholder engagement, CAR-T therapy, spinal muscular atrophy, evidence synthesis, EU HTAR

## Abstract

**Highlights:**

**What is the main finding?**

- This paper illustrates how systematic PICOS scoping can be operationalized within JCAs through Delphi consensus methods for mapping and reconciling multiple national and patient perspectives into a common framework.
- It highlights concrete examples of harmonization and clarifies the methodological tensions that arise when balancing rigor with flexibility.

**What are the implications of the main findings?**

- The findings underscore that early, structured stakeholder engagement—particularly involving patients—is essential for JCAs to be inclusive, scientifically robust, and relevant for national reimbursement decisions.
- Clear PICOS scoping could improve transparency while also enhancing the credibility and uptake of JCA outcomes in healthcare policy.

**Abstract:**

**Objectives:** This study explores the feasibility and value of integrating structured patient input into the PICOS (Population, Intervention, Comparator, Outcome, Study design) scoping process for Joint Clinical Assessments under the EU Health Technology Assessment Regulation. **Methods:** A modified Delphi panel, led by a steering committee composed of two clinicians, one patient expert, and one policy expert, engaged 12 individuals representing patient organizations across 12 European Member States to reach consensus on PICOS elements for CAR-T therapy in pediatric spinal muscular atrophy. **Results:** The Delphi process effectively facilitated PICOS consolidation and consensus among the 12 patient experts representing diverse EU contexts. Through 3 iterative rounds integrating quantitative rankings and qualitative feedback, the panel achieved strong agreement on key outcomes, intervention delivery, and study design elements, with population eligibility and comparator selection showing heterogeneity. Patient engagement was central: participants emphasized inclusive eligibility criteria, shared decision-making, and the inclusion of caregiver perspectives. The integration of qualitative insights allowed nuanced interpretation of dissent, distinguishing between genuine disagreement and framing effects, thereby enhancing transparency and scientific validity. Importantly, the process revealed patient priorities for outcomes, treatment burden, and evidence trade-offs, informing both PICOS refinement and future health technology assessment (HTA) strategies. This structured, participatory approach demonstrates the feasibility and value of incorporating patient voices systematically into early-stage EU HTA, fostering robust, credible, and context-sensitive consensus on complex rare-disease interventions. **Conclusions:** The study demonstrates the potential of consensus-building methodologies to enhance transparency, reduce heterogeneity, and support patient-centered evidence generation and decision-making in HTA.

## 1. Introduction

The implementation of the European Union (EU) Health Technology Assessment Regulation (HTAR) represents a significant step toward harmonizing the evaluation of health technologies across European Union Member States. Central to HTAR are the Joint Scientific Consultation (JSC) and the Joint Clinical Assessment (JCA), collaborative consultative and assessment processes grounded in the definition of the PICOS (Population, Intervention, Comparator, Outcome, Study design) elements that inform evidence generation and decision-making [1]. Yet the diversity of clinical practices, health system priorities, and patient needs across Europe presents challenges in designing unified and representative PICOS questions. To address these challenges, the HTAR requires each Member State to contribute to PICOS formulation through surveys coordinated at the EU level. While patients are recognized as key stakeholders in the regulation, their role in PICOS scoping is not explicitly defined.

We recently argued for a more inclusive and transparent approach to PICOS scoping for patients [2]. In our previous work, we proposed a novel theory-based, procedural framework for PICOS development under HTAR, advocating for consensus-based methods to engage a broad range of stakeholders—including patients and caregivers—early in the process to increase both the legitimacy and relevance of health technology assessment (HTA) outcomes across the EU [2].

This pilot study provides a case study of that framework in action. We employed a modified Delphi methodology to assess the feasibility and value of involving patient experts in PICOS development. The modified Delphi method, characterized by repeated survey rounds with controlled feedback, is a rigorous and efficient method to obtain consensus [3,4].

This study focused on a high-stakes emerging health technology, CAR-T therapy for pediatric patients with spinal muscular atrophy (SMA). SMA is a leading genetic cause of infant mortality and presents unique challenges in pediatric populations due to early onset, rapid progression, and variable severity. Recent advances have introduced disease-modifying therapies—gene replacement (onasemnogene abeparvovec), antisense oligonucleotides (nusinersen), and oral splicing modifiers (risdiplam)—alongside supportive care [5]. These options differ in administration, monitoring, and cost, creating complexity for HTA, especially given small trial populations and uncertainty around long-term outcomes.

Rare diseases such as SMA highlight the urgent need for tailored, patient-centered approaches to evidence generation and decision-making due to their complex clinical profiles and significant variation in patient preferences and outcomes across populations [6,7,8].

## 2. Objectives

This pilot study aimed to evaluate the feasibility, validity, and value of incorporating structured patient input into the early stages of HTA under HTAR. Specifically, the study had 3 objectives:

1. **Generate patient expert consensus on key PICOS elements** for CAR-T therapy in SMA, identifying areas of convergence and divergence with regard to evidence generation and assessment criteria.
2. **Explore the utility of the Delphi methodology for managing PICOS complexity** by supporting the consolidation of diverse input from patients and reducing heterogeneity in scoping under the JCA framework.
3. **Evaluate the operational feasibility and methodological robustness** of integrating structured patient insights into HTA evidence requirements, using a modified Delphi approach with quantitative and qualitative data to promote transparency, consistency, and scientific rigor.

## 3. Methods

### 3.1. Design

To meet the study’s objectives, a 3-round modified Delphi panel was designed and conducted jointly by OPEN Health and the European Patients’ Forum (EPF). Two physicians with expertise in SMA, one SMA patient representative, and one EU policy expert formed the steering committee. In this role, they provided input into study design, panel selection, survey development, and interpretation of results. In June 2024, the steering committee met virtually to discuss the research objectives and the content and structure of the consensus survey. Based on that discussion, a first draft of the survey was developed that consisted of consensus statements, ranking exercises, and open-ended questions for each PICOS domain. Prior to generating consensus statements, the steering committee reviewed existing HTA guidance and relevant literature on PICOS scoping and patient engagement. No formal systematic review was conducted; instead, evidence was summarized from prior publications and regulatory documents, which were shared with the steering committee to guide item development. The protocol for this study was not prospectively registered.

### 3.2. Panel Selection

EPF established a collaboration with the European Alliance of Neuromuscular Disorders Association (EAMDA). Twelve patients were recruited from EAMDA’s network in Europe. This sample size was deemed appropriate, given the rarity of SMA and the recommendation that a Delphi panel consist of 5–50 panelists [9]. Invited panelists were required to (1) be an SMA patient or a caregiver of an SMA patient involved with EAMDA or its national member societies through research, advocacy, or decision-making–related activities; awareness or knowledge of HTAR or PICOS scoping was not a prerequisite (an educational presentation was provided at the beginning of the interview round to provide a basic understanding of what HTAR and PICOS scoping implied) (2) have a good understanding of written and spoken English; and (3) be willing to provide their consent to participate in the study as a panelist, representing their specific network within the SMA patient community. Participants received financial remuneration for their time commitment and signed informed consent forms that outlined the nature of the study and the details of their involvement. Oral consent was also collected prior to the one-to-one interviews in Round 1.

### 3.3. Delphi Procedure

**Figure 1:**
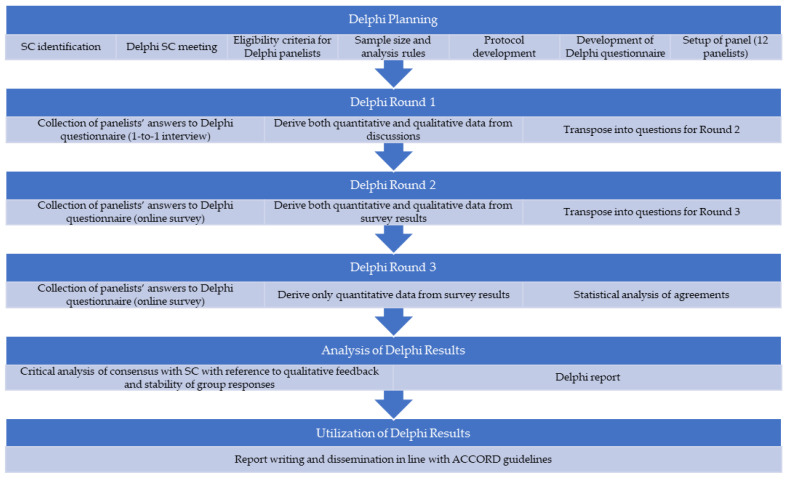
Study flow. Abbreviation: SC, steering committee.

The modified Delphi process followed a 3-round structure (Figure 1), which is well-supported in the literature as an optimal method to explore consensus in a timely and efficient way [4]. No piloting with panelists was conducted.

#### 3.3.1. Round 1

In Round 1, panelists were presented with 41 consensus statements, 2 ranking exercises, and 12 open-ended questions during one-to-one interviews with a member of the research team. All statements were answered on a 5-point Likert response scale (1 = Strongly Disagree, 2 = Disagree, 3 = Neutral, 4 = Agree, 5 = Strongly Agree). Interviews aimed to explore panelists’ preferences and insights related to each PICOS domain.

#### 3.3.2. Rounds 2 and 3

In Round 2, panelists were emailed a link to an electronic survey in Microsoft Forms with a total of 35 consensus statements and 5 open-ended questions. The survey included revised and new statements resulting from the analysis of the interviews conducted in Round 1. Following quantitative analysis of Round 2 results, 23 consensus statements and an additional 11 open-ended questions were brought forward to Round 3, the final round.

### 3.4. Analysis Rules, Process, and Reporting

This study followed established analysis guidance for Delphi studies, including those by De Loe et al. (1995) [3], Hasson et al. (2000) [4], Sprung et al. (2014) [10], Jünger et al. (2017) [11], and more recently Nurek et al. (2021) [12]. Based on this Delphi literature, a priori statistical definitions of consensus and dissensus were established:

- Consensus was defined as a minimum of 80% agreement across the contiguous Likert categories “Agree” and “Strongly Agree.”
- Dissensus was defined as ≥80% disagreement across the contiguous Likert categories “Disagree” and “Strongly Disagree.”

In addition to consensus and dissensus, statements were further categorized by level of agreement at the end of Round 3:

- High level of agreement without consensus: 60–80% of panelists agreed (“Agree” + “Strongly Agree”).
- Low level of agreement: <60% agreement.

As recommended by Vogel et al. (2019), responses of “I don’t know/Cannot answer” were not counted toward consensus calculations to ensure that consensus levels reflected only the input of those who felt confident responding to a given statement [13]. Participants who selected “I don’t know” were invited to provide qualitative comments to contextualize their uncertainty.

All response frequencies and qualitative feedback were analyzed using a combination of descriptive statistics and thematic content analysis. Suggested revisions to statements were incorporated when proposed by at least 2 panelists and reviewed by the steering committee for inclusion in the next round. The entire set of analysis rules (Table 1) was standardized and approved in advance by the steering committee and used consistently across all rounds.

To ensure transparency and methodological rigor, the design, conduct, and reporting of the Delphi consensus process followed the ACCORD (Accurate Consensus Reporting Methods) reporting framework [14] (see Section S6 in Supplementary Materials). This included clear documentation of panel composition, consensus criteria, number of rounds, methods for statement refinement, and procedures for integrating qualitative and quantitative data. The application of ACCORD ensures that the consensus findings are robust, reproducible, and aligned with best-practice standards for stakeholder engagement and consensus development.

## 4. Results

### 4.1. Panel Composition

**Figure 2:**
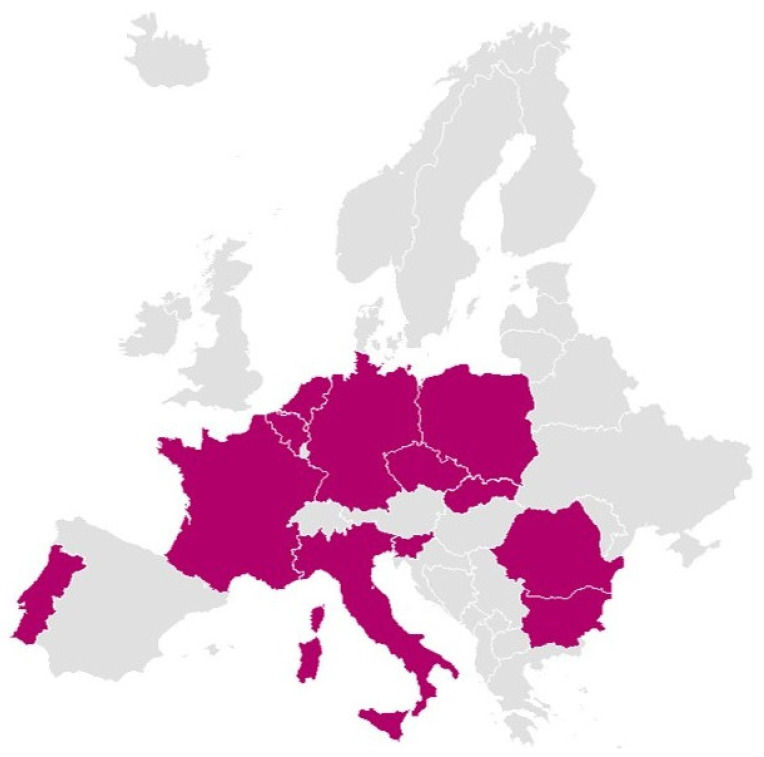
Countries Represented by the Panelists.

The Delphi panel included 12 patient experts, including both actual patients and caregivers, representing SMA communities from 12 different EU Member States, representative of the variety of geographies, country sizes, per capita income, and healthcare systems that exist across the EU. All participants were actively engaged in national and European patient advocacy groups, with 89% reporting access to patient communities of 50–200 individuals and the remaining 11% to smaller networks. All panelists confirmed a high level of comfort with representing the voice of SMA patients in their respective countries. Figure 2 illustrates the countries represented.

### 4.2. Consensus Outcomes

Consensus evolved progressively across the 3 rounds, with certain domains—particularly Outcomes, Intervention, and Study design—achieving higher agreement levels earlier in the process. In contrast, responses in the Population and Comparator domains initially showed more heterogeneity, reflecting diverse national contexts and patient experiences. Nonetheless, the structured feedback and incorporation of qualitative insights allowed for significant alignment over the course of the study. A summary of the results of the consensus exercise is provided in Table 2. See the Supplementary Materials for a detailed overview of the statements that reached consensus across each round and those that did not, an overview of all statements that reached consensus across each round, and an overview of all statements that did not reach consensus across each round.

#### 4.2.1. Population

Consensus emerged in favor of adopting broader, more inclusive eligibility criteria for CAR-T therapy. Panelists strongly supported including pre-symptomatic patients, such as those identified through newborn screening, and patients of any age if they met genetic diagnostic criteria. Importantly, panelists advocated for moving beyond strict clinical trial inclusion criteria, which were seen as overly restrictive and not always reflective of real-world clinical needs. The panel also conveyed strong support for shared decision-making frameworks, wherein eligibility would be determined collaboratively by clinicians, patients, and caregivers, taking into account individual patient profiles, preferences, and values. Dissent remained around whether motor and respiratory function thresholds should guide access to treatment and how closely the thresholds should mirror those in existing trial protocols.

#### 4.2.2. Intervention

There was early and sustained agreement on the need for a comprehensive and specialized approach to CAR-T therapy delivery. Panelists emphasized the importance of care being provided in established centers with experienced staff, preceded by robust pre-treatment screening and followed by active monitoring and caregiver support. Equitable access across Member States was a recurring theme, alongside the need to manage treatment burden, particularly in relation to corticosteroid use and administration logistics. While consensus was reached on most aspects of intervention delivery, disagreement persisted around preference between CAR-T and other treatment modalities, administration routes (e.g., spinal vs. oral), and the consideration of indirect costs, such as caregiver burden and out-of-pocket expenses, in HTA decision-making.

#### 4.2.3. Comparator

Consensus was achieved on the inclusion of best supportive care and no active treatment as essential baseline comparators. There was also broad support for integrating patient and caregiver preferences into the selection of additional comparators. However, agreement was not reached on the selection of active comparators, due to the high variability of patient priorities and attitudes toward uncertainty, risks, and outcomes across different national and clinical settings. This divergence underscores the need for HTA frameworks to accommodate personal patient preferences and allow for context-sensitive comparator choices.

#### 4.2.4. Outcomes

A high level of agreement was reached on the importance of motor function improvement, survival without permanent ventilation, and the achievement of age- and subtype-specific motor milestones as primary outcomes. The panel also supported including quality-of-life measures for both patients and caregivers as critical endpoints. Furthermore, participants called for the development of more sensitive and specific outcome measurement tools, particularly to detect subtle but meaningful changes in function. Some divergence was observed around the relative weight of caregiver quality of life versus patient-centric outcomes, reflecting differing priorities between stakeholder roles.

#### 4.2.5. Study Design

Panelists strongly supported a multimodal approach to evidence generation, favoring the inclusion of randomized controlled trials (RCTs) and real-world evidence (RWE) that includes patient-reported outcomes, along with single-arm trials. This reflects an understanding of the evidentiary limitations in rare diseases such as SMA and the value of triangulating different data types to inform HTA. There was consensus on the importance of broadening access to clinical trials across the EU and allowing previously treated patients to participate in future research. Divergence remained regarding the trade-off between evidentiary robustness and geographic accessibility, with some panelists prioritizing access to innovative therapies even if it meant accepting less mature data.

### 4.3. Findings from Round 1 Ranking Exercises and Qualitative Data

In Round 1, participants completed structured ranking exercises to assess priorities for CAR-T therapy in SMA, including (1) outcome preferences and (2) willingness to trade off evidence certainty for earlier access. These exercises were complemented by semi-structured interviews and open-ended responses that were retained in Rounds 2 and 3, providing critical context and depth to the quantitative answers. Full details of ranking results and qualitative coding themes are available in the Supplementary Materials.

Together, the rankings and qualitative insights laid the foundation for guiding consensus development. Rankings offered a baseline of shared priorities, while qualitative data revealed underlying values, rationales, and areas of divergence, informing the refinement of statements for Rounds 2 and 3.

On outcome preferences, participants consistently prioritized survival, motor function, and respiratory outcomes, with more variation around quality-of-life measures. Interviews and qualitative data clarified that lower rankings did not reflect disinterest but rather prioritization of life-saving effects. Differences between patients and caregivers—the former emphasizing autonomy, the latter emotional burden—highlighted the need to accommodate diverse perspectives within HTA processes, particularly when different therapeutic options are available.

On evidence-access trade-offs, most participants favored robust evidence over early access, citing the availability of existing SMA therapies. Openness to uncertainty was more common in countries with limited access and was highly outcome-specific. These insights helped explain areas of dissensus, particularly around comparators and caregiver priorities.

Qualitative feedback across rounds also revealed key themes: support and characteristics for shared decision-making, frustration with rigid eligibility criteria, concerns about information gaps, and criticism of narrow and outdated outcome measures. Participants called for more holistic, patient-centered metrics and clearer communication around treatment expectations.

The integration of both ranking and qualitative data was central to refining and expanding the Delphi statements. Insights led to the introduction of new items on caregiver burden, information needs, indirect costs, and trial accessibility, and informed the rewording of statements to better reflect patient values and context-specific concerns. Crucially, the qualitative input guided interpretation of dissensus, allowing the research team to distinguish between true disagreement and differences driven by framing or misunderstanding. The inclusion and analysis of free-text responses is consistent with best practice in consensus research, particularly in rare disease settings, where heterogeneity of experience can otherwise be masked by quantitative aggregation alone [4].

This iterative, mixed-methods approach ensured that the Delphi process was not only statistically grounded but also responsive to lived experience, making the resulting consensus credible, relevant, and representative.

## 5. Discussion

This pilot Delphi study underscores the feasibility and value of using structured consensus methods to involve patient experts in the early stages of health technology assessment, particularly in the complex, cross-national context of the EU HTAR. The inclusion of patient voices through a 3-round Delphi process enabled not only the identification of shared priorities, but also a systematic exploration of dissent and diversity in views across different Member States.

The results demonstrate that while consensus is achievable on key elements—such as the need for broader inclusion criteria, caregiver support, and diverse forms of evidence—significant heterogeneity remains. This heterogeneity is not a weakness of the method, but a feature of pluralistic and democratic health policymaking. By making these divergences visible and traceable, the Delphi method strengthens transparency and accountability.

These findings might have important implications for appraisal and payer decision-making. While patients strongly favored broader eligibility criteria and inclusion of real-world evidence, many payers traditionally restrict reimbursement to trial-defined populations. Extending eligibility may therefore be difficult to achieve without robust supporting evidence. Structured patient input, as demonstrated in this study, can inform early dialog and help identify areas where flexibility is most needed, supporting more transparent and patient-centered decisions.

From a methodological standpoint, the inclusion of open-ended interviews in Round 1 enriched the process by allowing for nuanced framing of statements in later rounds. This adaptation—supported by previous methodological research [4]—proved particularly beneficial in capturing the complexity of patient preferences in rare disease contexts. The combination of statistical consensus thresholds and thematic qualitative analysis allowed for a comprehensive interpretation and actionable insights.

JCAs under HTAR impose technical constraints such as the need to consolidate PICOS and reliance on direct evidence from randomized trials. These requirements often conflict with patients’ diverse preferences and need for more comprehensive evidence packages that include real-world evidence and patient-reported outcomes. In addition, regulatory constraints—such as strict timelines and the need to manage potential conflicts of interest among diverse stakeholders—further limit opportunities for iterative, inclusive input. Consensus-based approaches like Delphi panels may offer a structured, transparent way to navigate these challenges by systematically integrating patient perspectives into PICOS scoping while maintaining methodological rigor and traceability, helping reconcile regulatory demands with stakeholder diversity.

This study has several limitations. First, the absence of clinicians limited the triangulation of patient perspectives with medical expertise. Future work should explore whether joint or parallel Delphi processes with healthcare professionals improve the balance and usability of outcomes.

Second, the method was resource- and time-intensive, which may limit scalability under tight JCA timelines. While participants represented multiple EU countries, pan-European representativeness remains challenging due to disparities in advocacy capacity and healthcare systems, and the requirement for proficiency in English, which may have introduced a selection bias.

Third, the use of a 5-point Likert scale may introduce central tendency bias, where respondents disproportionately select the neutral midpoint option, potentially masking true variation in opinions. This phenomenon is well-documented in survey research and can affect the reliability and validity of results [15,16]. Additionally, following Vogel et al. (2019) [13], “I don’t know/Cannot answer” responses were excluded from consensus calculations to ensure that agreement reflected informed judgments. While this approach is methodologically recommended, it may introduce bias by omitting uncertainty, particularly among less-informed participants. In our case, the impact was minimal: only 7 statements in Round 1 (with a maximum of 3 “I don’t know/Cannot answer” responses per statement) and 4 statements in Round 3 (with a maximum of 2 “I don’t know/Cannot answer” responses per statement) contained these responses across all rounds. However, future studies should consider alternative strategies, such as weighting uncertain responses or analyzing them qualitatively.

Fourth, because CAR-T therapy for SMA involves a rare disease setting with limited treatment alternatives, this study could not demonstrate whether consensus input might help consolidate and prioritize comparator preferences and reduce the number of comparators from a patient perspective, a function that may be more relevant in therapeutic areas with multiple competing interventions. Broader application in more crowded therapeutic areas is needed to test this function.

Fifth, although the panel included representatives from multiple EU Member States, Scandinavian countries were not represented. This might represent a limitation, as these countries have distinct tax-funded healthcare systems and a strong tradition of patient involvement, which may influence PICOS priorities. Future studies should aim to include these regions to ensure broader representativeness.

Despite these limitations, this pilot shows that consensus research can directly address EU HTAR challenges by supporting transparent, inclusive, and comprehensive PICOS scoping. The Delphi method offers a structured approach to engage stakeholders across Member States and manage divergent inputs in a consistent way. This exercise revealed relevant outcomes—such as patient preferences for comparators and perceptions over measurement tools—that could strongly shape evidence generation strategies and assessment practices, particularly if performed during JSCs.

Beyond this, consensus methods can help build the infrastructure for long-term stakeholder engagement in Europe. As HTA processes become more integrated, these approaches can support the creation of a more equitable and methodologically sound EU healthcare system, where patient and caregiver input informs both science and policy.

## 6. Conclusions and Key Considerations

This pilot Delphi study demonstrates that structured patient consensus methods can meaningfully inform the PICOS scoping process under HTAR. By capturing diverse patient perspectives through a rigorous, iterative approach, this method offers a scalable and credible model for embedding patient input into JCA.

The study shows that patient-informed consensus can enhance both the scientific quality and legitimacy of HTA by ensuring that differences in clinical practice, access, and values across Member States are reflected in a coherent and inclusive way. Consensus on broader inclusion criteria, shared decision-making, and support for real-world evidence provides actionable input for future trial design and JCA planning. Areas of divergence—such as comparator preferences and caregiver outcomes—underscore the need for continued stakeholder dialog and national-level adaptation.

Future work should include clinicians to assess whether joint or parallel Delphi panels with patients and professionals yield better outcomes. This approach has the potential to strengthen EU HTA by grounding assessments in both evidence and lived experience, supporting a more unified, responsive European healthcare system.

## Figures and Tables

**Table 1:** Analysis Rules Used in the Modified Delphi.

**Rule 1**
Statements that receive < 60% agreement (“Agree” and “Strongly Agree”) will be considered as having “low agreement”
**Rule 2**
Statements that receive 60–80% agreement (“Agree” and “Strongly Agree”) will be considered as having “medium agreement”
**Rule 3**
Statement whose sum of its agreement response frequencies across two contiguous categories (i.e., “Agree” and “Strongly Agree”) is ≥80% will be considered “Consensus” and will not be presented in the subsequent round
**Rule 4**
Statements whose sum of disagree response frequencies across two contiguous categories (i.e., “Disagree” and “Strongly Disagree”) is ≥80% will be considered “Dissensus” and will not be asked in the subsequent round
**Rule 5**
“I don’t know” responses will not be used to calculate percentage frequencies to determine consensus, to ensure that consensus is calculated only amongst those who knew the answer. “I don’t know” responses will also be excluded when calculating the level of agreement for statements that did not reach consensus
**Rule 6**
For qualitative comments suggesting that a statement should be edited, at least 10% of the panelists will need to provide the same suggestion before an edit is made

**Table 2:** Summary of Delphi Results. EU indicates European Union; PRO, patient-reported outcome; RCT, randomized controlled trial; RWE, real-world evidence.

PICOS	Round 1	Round 2	Round 3	Final Results	Topics of Consensus
**Population**	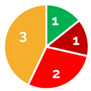	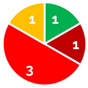	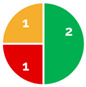	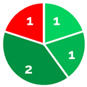	(1) Broader inclusion criteria; (2) Pre-symptomatic patients; (3) Need to expand beyond trial criteria; (4) Need for clear shared decision-making tools
**Intervention**	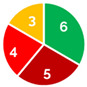	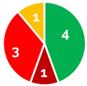	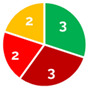	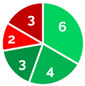	(1) Need for specialized care; (2) Pre-treatment planning & Caregiver support; (3) Corticosteroid burden management; (4) Equitable access
**Comparator**	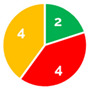	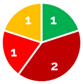	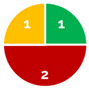	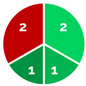	(1) Acceptance of no active treatment; (2) Need to include best supportive care; (3) Include patient/caregiver input; (4) Harmonized information for decision-making
**Outcomes**	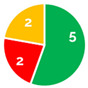	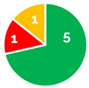	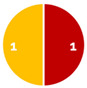	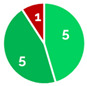	(1) Prioritize survival and motor function; (2) Focus on patient/caregiver-reported outcomes; (3) New sensitive measurement tools; (4) Trial access across EU
**Study Design**	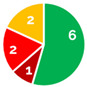	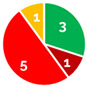	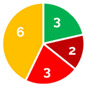	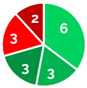	(1) Need for RCTs + RWE + PROs + Single-arm trials; (2) Broad EU participation; (3) Include previously treated patients in studies

## Data Availability

The data supporting the findings of this study are not publicly available due to privacy and ethical restrictions related to participant confidentiality. De-identified excerpts or summaries of the qualitative data may be made available from the corresponding author upon reasonable request and with permission from the participants.

## Supplementary Materials

The following supporting information can be downloaded at: https://www.mdpi.com/article/10.3390/jmahp14010006/s1, Section S1: Delphi Statements Across Rounds; Section S2: Delphi Statements That Reached Consensus; Section S3: Delphi Statements That Did Not Reach Consensus; Section S4: The Role of the Ranking Exercises; Section S5: Qualitative Insights; Section S6: ACCORD Guidelines.
