# Supplementary material for: Enhancing Patient-Centered Health Technology Assessment: A Modified Delphi Panel for PICOS Scoping in Spinal Muscular Atrophy
Source: J Mark Access Health Policy. 2026 Jan 19;14(1):6. doi: 10.3390/jmahp14010006 (PMC12922062; doi:10.3390/jmahp14010006)
Supplement: Supplementary file 1 [file jmahp-14-00006-s001.zip › jmahp-3957257-supplementary.pdf]

Supplementary Material

Enhancing Patient-Centered Health Technology Assessment: A Modified Delphi Panel for PICOS Scoping in Spinal Muscular Atrophy

Section S1: Delphi Statements Across Rounds

Round 1 Statements

	Statements	Consensus reached (Yes/No)	Agreement Score
Population			*% agreement, % disagreement, I don't know**
1	The group of patients that receive Zolgensma should exclusively be decided by specialised medical doctors that rely their decision on clinical evidence	No	75%;25%;0
2	The group of patients that receive Zolgensma should include pre-symptomatic patients identified through newborn screening.	Yes	91%;9%;0
3	The group of patients that receive Zolgensma should include symptomatic patients, across all SMA types, identified through genetic testing, but the inclusion criteria should focus on age, weight, previous treatment, motor function, and comorbidity.	No	67%;17%;0
4	Zolgensma should be available exclusively to patients that fit the inclusion criteria used in the SMART study, the main trial evaluating Zolgensma use in children up to 9 years old who have been diagnosed with SMA, confirmed by a mutation in the SMN1 gene, that weight up until 21 kg and regardless of motor function.	No	33%;58%;0
5	Do you think Zolgensma should be available for other groups of SMA patients other than the ones included in the main trial (SMART study)?	-	-
6	In your opinion, are there other important patient's characteristics that should be considered when deciding who should have access to Zolgensma?	-	-
Intervention			*% agreement, % disagreement, I don't know**
7	How a treatment works, its formulation, dosing, side effects, and how it is given are all factors that influence patient preference for the type of intervention we receive.	No	75%;0%;0
8	A treatment like Zolgensma, which works by fixing the genetic problem causing SMA, is preferrable to a treatment that focuses on treating the symptoms and slowing the progression of the disease.	No	67%;17%;0

9	The administration of Zolgensma, which requires a single intravenous infusion (IV) at the hospital, is preferred to an oral treatment, taken daily at home, for life.	No	55%;18%;0
10	The single, intravenous infusion of Zolgensma should be administered in a specialized clinic or hospital, by a healthcare professional with experience in SMA.	Yes	92%;0%;0
11	The single, intravenous infusion of Zolgensma should be administered in a specialized clinic or hospital, by a healthcare professional with experience in gene therapy.	No	75%;8%;0
12	A pharmacological treatment plan for SMA should be comprehensive of pre-treatment screening to confirm diagnosis, dosing regimen based on patients' characteristics (e.g. age, weight, previous treatment, social context), and best supportive care.	Yes	100%;0%;0
13	After the administration of Zolgensma, it's important to have regular check-ups to keep track of patient's health and address any side effects that might happen.	Yes	100%;0%;1
14	Are there other aspects of post-infusion care that you think are important for a patient?	-	-
15	Support to caregivers of a patient with SMA, such as emotional and educational support, is important to maximise the treatment's benefits for the patient and manage any challenge caused by the treatment and/or the condition.	Yes	100%;0%;0
16	Are there other types of support that you think could benefit the caregiver?	-	-
17	Although they help with gene therapy, treatment with corticosteroids can be challenging as they can cause side effects (such as bone weakness, delayed growth, weight gain) that add an additional burden to the patient and their caregiver.	Yes	82%;0%;1
18	The additional burden posed by the side effects of corticosteroids, which are often used alongside gene therapy, should be considered in the overall evaluation of gene therapy.	Yes	90%;0%;2
19	SMA patients experience difficulties in accessing gene therapy treatment in my country.	No	17%;50%;0
20	Getting to the treatment centre for gene therapy can be a big challenge for some SMA patients and their caregivers in my country.	No	25%;75%;0
21	Accessing gene therapy can be challenging in my country because not all healthcare providers (HCPs) are familiar with it.	No	33%;58%;0
22	In my country, treatment for SMA carries significant indirect costs, such as reduced working time and transportation, for patients and their families.	No	75%;25%;0
23	The indirect costs of SMA treatment should be considered in the overall evaluation of gene therapy.	No	50%;50%;0
Comparator			*% agreement, % disagreement, I don't know**
24	The right comparator for Zolgensma should exclusively be decided by specialized medical doctors that rely their decision on clinical evidence.	No	50%;42%;0

25	Nusinersen, administered via intrathecal injection with a loading dose followed by maintenance doses every four months, is the appropriate comparator for SMA patients.	No	50%;33%;0
26	Risdiplam, administered as a daily oral solution based on weight, is the appropriate comparator for SMA patients.	No	50%;33%;0
27	Best supportive care including physical therapy, respiratory support, and nutritional support should be assessed in addition to any relevant comparator for SMA patients.	Yes	92%;8%;0
28	No treatment, including historical control groups or untreated cases, could also be considered the appropriate comparator for SMA patients.	Yes	90%;10%;2
29	Patients receive sufficient information (including potential risks and benefits) about the different treatment options for SMA in my country.	No	50%;50%;0
30	Are there any specific treatments or interventions that you feel should be included as a comparator?	-	-<br>-
31	Are there any specific outcomes or side effects that you would like to see compared between the intervention and the comparator?	-	-
32	What factors are most important to you when considering a comparator?	-	-
Outcomes			*% agreement, % disagreement, I don't know**
33	Primary outcomes should include improvement in motor function (e.g., CHOP-INTEND score, Hammersmith Functional Motor Scale, RULM score, ability to stand and walk).	Yes	92%;0%;0
34	Primary outcomes should include survival without permanent ventilation (particularly for SMA Type 1).	Yes	100%;0%;1
35	Secondary outcomes should include achievement of motor milestones (e.g., sitting unassisted for SMA Type 1, standing and walking for SMA Type 2)	No	75%;0%;0
36	Secondary outcomes should include respiratory function and independence from respiratory support.	Yes	100%;0%;0
37	Secondary outcomes should include quality of life improvements for patients such as physical abilities (incl. pain and fatigue), emotional well-being, social participation, and overall daily living activities.	Yes	92%;0%;0
38	Secondary outcomes should include quality of life improvements for caregivers such as emotional well-being, social participation, reduced burden and access to resources.	No	75%;8%;0
39	Secondary outcomes should include adverse events and safety profile.	Yes	100%;0%;0
40	Outcomes- ranking	-	-
Study design			*% agreement, % disagreement, I don't know**
41	Randomized controlled trials (RCTs), comparing Zolgensma to another treatment, are the gold standard for assessing the efficacy and safety of Zolgensma.	Yes	83%;17%;0
42	Randomized controlled trials (RCTs), comparing Zolgensma to another treatment must be conducted in EU countries.	No	33%;67%;0

43	Randomized controlled trials (RCTs), comparing Zolgensma to another treatment must allow patients to switch arm during the trial.	No	78%;0%;3
44	Single-arm trials, where all participants receive Zolgensma, are the best method for understanding the treatment's effects from a patient point of view, but should include children from a broad range of EU countries.	No	70%;10%;2
45	Real-world evidence (RWE) studies are essential for capturing the long-term effectiveness and safety of Zolgensma in everyday clinical practice and should be an important part of the evidence package.	Yes	100%;0%;0
46	Real-world evidence (RWE) studies should involve patients across a broad range of EU countries, using data from clinical records, patient registries, and other real-world sources.	Yes	92%;8%;0
47	Patient-reported outcomes (PRO) studies are essential for understanding the impact of Zolgensma on quality of life from the patient's and caregiver's perspectives and should be an important part of the evidence package.	Yes	100%;0%;0
48	Patient-reported outcomes (PRO) studies should include patients from a broad range of EU countries and should assess patients inputs and preferences on factors such as physical abilities, emotional well-being, social participation, and daily living activities.	Yes	92%;8%;0
49	Patients are open to incorporate insufficient evidence from traditional study designs (such as RCTs) with innovative research methods (such as Single-arm trials, RWE and PRO research) that might provide useful in providing additional evidence for decision-making.	Yes	91%;0%;1
50	Study design- ranking	-	-

***% Agreement** = Sum of "Agree" + "Strongly Agree" responses; **% Disagreement** = Sum of "Disagree" + "Strongly Disagree" responses; **I Don't Know** = Number of "I don't know" responses

Round 2 Statements

	Statements	Consensus reached (Yes/No)	
Population			*% agreement, % disagreement, I don't know**
1	The group of patients that receive Zolgensma should exclusively be decided by specialised medical doctors (i.e. a neurologist) that rely their decision on clinical evidence and in consultation with patients and/or their caregivers.	Yes	90%;10%;0
2	The group of patients with SMA that receive Zolgensma should include symptomatic patients, across all SMA types, regardless of age, identified through genetic testing, and meet the inclusion criteria for weight, previous treatment, and motor function.	Yes	78%;22%;0
3	Zolgensma should be available to patients that fit the inclusion criteria used in the SMART study; the main trial evaluating Zolgensma use in children who have been diagnosed with	No	80%;10%;0

	SMA, confirmed by a mutation in the SMN1 gene, and weigh up to 21 kg.		
4	Age should not be a restrictive factor for the selection of patients that can receive Zolgensma, although individual patient preferences should be considered.	No	60%;30%;0
5	The selection of patients for Zolgensma treatment should consider the status of their motor and respiratory functions. These functions should not be severely impaired and there should still be the possibility to gain meaningful improvements.	No	40%;60%;0
Intervention			*% agreement, % disagreement, I don't know**
6	The effect of treatment, its safety, formulation, dosing, administration characteristics (e.g. one or multiple shots), the burden of treatment for the patient, patient's comorbidity(ies), are all factors that influence patient preference for the type of intervention we receive.	Yes	100%;0%;0
7	A treatment like Zolgensma, which aims at delivering a functional copy of the SMN1 gene, improving motor function, and achieving developmental milestones, is generally preferable to a treatment that works by targeting the SMN2 gene production of SMN protein and aims at treating the symptoms and slowing the progression of the disease.	No	40%;10%;0
8	The administration of Zolgensma, which requires a single intravenous infusion (IV), is preferred to a treatment that requires multiple spinal injections (typically, 4 injections over the first two months, followed by a maintenance injection every 4 months, for life).	No	50%;20%;0
9	The administration of Zolgensma, which requires a single intravenous infusion (IV) at the hospital, is preferred to an oral treatment, taken daily at home, for life.	No	40%;30%;0
10	For the delivery of a gene therapy, it is more important to have healthcare professionals with experience in SMA and IV infusions than healthcare professionals with experience in gene therapy.	No	70%;20%;0
11	Patient support programs and availability of information for patients and their caregivers should be evaluated as part of the intervention characteristics.	Yes	100%;0%;0
12	Support to caregivers such as the availability of information regarding different treatment options and financial support are important to maximise the treatment's benefits for the patient and manage any challenge caused by the treatment and/or the condition.	Yes	100%;0%;0
13	In my country, patients with SMA have access to gene therapy provided by experienced doctors.	Yes	90%;10%;0
14	There are sufficient centres offering gene therapy in my country, and they are adequately distributed across the country.	No	70%;10%;0
15	The treatment and follow-up care for patients undergoing gene therapy impose significant indirect costs on parents and caregivers, primarily due to time away from work which may negatively impact their salaries.	No	60%;0%;0
16	It is not necessary to assess the indirect costs of SMA gene therapy treatment for patients and caregivers in the evaluation of the therapy.	No	0%;67%;0

Comparator			
17	Patients and their families should be involved in deciding the right comparator for Zolgensma along with specialised medical doctors.	Yes	100%;0%;0
18	Nusinersen which has a different mechanism from Zolgensma, and is administered every four months, is not a relevant comparator for patients with SMA because these products are used across different patient groups and for different purposes.	No	33%;56%;0
19	Risdiplam, which has a different mechanism from Zolgensma, and is administered as a daily oral solution based on weight, is not a relevant comparator for patients with SMA because they are used across different patient groups and for different purposes.	No	33%;56%;0
20	Patients' and caregivers' preferences on treatment outcomes such as the treatment impact on patients' Quality of Life, play a vital role in choosing the right comparator.	Yes	80%;20%;0
21	Treatment outcomes preferences, such as impact on Quality of Life, play a vital role in choosing the right comparator.	Yes	100%;0%;0
Outcomes			
22	Primary outcomes should include achievement of motor milestones (e.g., sitting unassisted for SMA Type 1, standing and walking for SMA Type 2).	Yes	90%;0%;0
23	Quality of life of the caregivers is an important secondary outcome and should be considered.	Yes	90%;0%;0
24	Patients and caregivers in my country generally prioritise overall survival and motor skills improvement, followed by improvement in respiratory abilities, survival without long-term ventilation, physical and emotional quality of life, and the safety profile. Less priority is given to caregiver quality of life and access to support resources	No	60%;20%;0
25	Patients' and caregivers' priorities differ based on their SMA type and patient's characteristics. For instance:<br>infants with SMA Type 1 may prioritize on overall survival and improvement in respiratory abilities, as these patients are at high risk for severe respiratory complications;<br>children with SMA Type 2 may prioritize motor skills improvement, such as achieving unassisted sitting or standing, and survival without long-term ventilation, as these milestones significantly impact their independence and quality of life;<br>adolescents and adults with SMA Type 3 may prioritize enhancing physical quality of life and reducing pain and fatigue, as they may already have some degree of motor function but struggle with daily living activities.	Yes	100%;0%;0
26	There is a need to develop new scales that are able to capture more granular improvements in motor skills due to the lack of sensitivity of current scales to measure small progresses.	Yes	100%;0%;0
Study design			*% agreement, % disagreement, I don't know**
27	Ideally, Randomised controlled trials (RCTs), comparing Zolgensma to another treatment are the gold standard for assessing the efficacy and safety of Zolgensma.	No	60%;10%;0

28	Patients are generally comfortable with data from RCTs, regardless of where they are conducted globally, provided that the highest standards of quality and ethical conduct are upheld.	No	70%;10%;0
29	Patients believe it is important to conduct RCTs within Europe to ensure they have the opportunity to participate in trials across the region, regardless of the specific country where the research is conducted.	Yes	89%;0%;0
30	Single-arm trials, where all participants receive Zolgensma, are an acceptable method for understanding the treatment's effects from a patient point of view.	No	70%;10%;0
31	Patients believe it is important to conduct Single-arm trials within Europe to ensure they have the opportunity to participate in trials across the region, regardless of the specific country where the research is conducted.	No	70%;10%;0
32	Patients and caregivers are open to incorporate insufficient evidence from traditional study designs (such as RCTs or Single-arm trials) with innovative research methods (such as RWE and PRO research) that might provide additional evidence for decision-making.	Yes	100%;0%;0
33	In my country, patients (or their caregivers) would prefer moderately accessible innovative treatment such as Zolgensma (available within average timelines and/or average costs) with moderate certainty (some evidence about how well it works, but not fully proven).	No	38%;25%;0
34	In my country, patients (or their caregivers) would prefer less accessible innovative treatment such as Zolgensma (wait longer for evidence generation and/or more costly) with higher certainty (strong evidence that it works well).	No	67%;11%;0
35	New SMA trials, including those for gene therapies, are often limited to treatment-naive patients (patients that did not receive any previous treatment for SMA). This poses a significant issue because most patients are already on SMN-targeted therapies. It would be more beneficial if these trials were designed to also include patients who already had previous treatments.	Yes	90%;0%;0

***% Agreement** = Sum of "Agree" + "Strongly Agree" responses; **% Disagreement** = Sum of "Disagree" + "Strongly Disagree" responses; **I Don't Know** = Number of "I don't know" responses

Round 3 Statements

	Statements	Consensus reached (Yes/No)	
Population			*% agreement, % disagreement, I don't know**
1	As a minimum standard, Zolgensma should be available to patients that fit the inclusion criteria used in the SMART study; the main trial evaluating Zolgensma use in children who have been diagnosed with SMA, confirmed by a mutation in the SMN1 gene, and weigh up to 21 kg.	Yes	100%;0%;0
2	A patient's age alone should not determine their eligibility for Zolgensma treatment.	Yes	100%;0%;0

3	When selecting patients for Zolgensma treatment, it's important to consider their motor and respiratory functions. While these functions should ideally not be severely impaired, patients with significant respiratory issues should not be automatically excluded, as any improvement can be meaningful for them.	No	67%;8%;0
Intervention			
4	A treatment like Zolgensma, which aims at delivering a functional copy of the SMN1 gene, improving motor function, and achieving developmental milestones, is generally preferable to a treatment that works by targeting the SMN2 gene production of SMN protein and aims at treating the symptoms and slowing the progression of the disease. However, the choice of treatment should ultimately be made on an individual basis, taking into account personal preferences and characteristics.	No	67%;17%;0
5	The administration of Zolgensma, which requires a single intravenous infusion (IV), is generally preferred over treatments that require multiple spinal injections (typically, 4 injections over the first two months, followed by a maintenance injection every 4 months for life). However, the choice of treatment should ultimately be made on an individual basis, taking into account personal preferences and characteristics.	No	67%;25%;0
6	The administration of Zolgensma, which requires a single intravenous infusion (IV) at the hospital, is preferred to an oral treatment, taken daily at home, for life. However, the choice of treatment should ultimately be made on an individual basis, taking into account personal preferences and characteristics.	No	50%;17%;0
7	Ideally, gene therapy administration for SMA should involve a multidisciplinary team comprised of healthcare professionals experienced in both SMA and gene therapies.	Yes	100%;0%;0
8	Access to healthcare centres offering gene therapy varies significantly across European countries. While some countries have sufficient number of centres with good access, other countries face poor access and limited availability of centres.	Yes	100%;0%;0
9	In some cases, the treatment and follow-up care for patients undergoing gene therapy impose significant indirect costs on parents and caregivers, primarily due to time away from work which may negatively impact their salaries.	Yes	92%;0%;0
10	The indirect costs associated with SMA gene therapy should be factored into the overall assessment of the treatment.	No	58%;17%;0
11	For patients receiving SMA gene therapy, including indirect costs in drug assessments is important, whereas caregivers place less emphasis on these costs	No	55%;9%;0
Comparator			*% agreement, % disagreement, I don't know**
12	Nusinersen which has a different mechanism from Zolgensma, and is administered every four months, is not a relevant comparator for patients with SMA because these products are used across different patient groups and for different purposes.	No	33%;50%;0

13	Risdiplam, which has a different mechanism from Zolgensma, and is administered as a daily oral solution based on weight, is not a relevant comparator for patients with SMA because they are used across different patient groups and for different purposes.	No	33%;50%;0
14	Patients and caregivers should receive high-quality, harmonized and comprehensive information on the risks and benefits of different treatment options for SMA in my country to make an informed decision.	Yes	100%;0%;0
Outcomes			*% agreement, % disagreement, I don't know**
15	When evaluating a therapy for SMA, patients and caregivers typically prioritise aspects like overall survival and motor skills improvements over the caregiver's quality of life and access to support resources.	No	50%;42%;0
Study design			*% agreement, % disagreement, I don't know**
16	Patients are generally comfortable with data from Randomized Control Trials, regardless of where they are conducted, provided that the highest standards of quality and ethical conduct are upheld.	No	58%;25%;0
17	Single-arm trials, where all participants receive the same treatment (Zolgensma), with no comparison group or placebo, are an acceptable method for understanding the treatment's effects from a patient point of view.	Yes	100%;0%;0
18	It is important to conduct single-arm trials within Europe to ensure that Europe-based patients have the opportunity to participate in trials, regardless of the specific country where the research is conducted.	Yes	83%;17%;0
19	Patients may choose to stay close to family rather than traveling for the most effective treatment, especially when multiple options are available, even if less effective.	No	73%;18%;1
20	In general, patients (or their caregivers) prefer innovative treatments like Zolgensma, even if they are less accessible due to higher costs or due to the need for more evidence.	No	75%;17%;0
21	Randomised controlled trials (RCTs), comparing Zolgensma to another treatment are the gold standard for assessing the efficacy and safety of Zolgensma.	Yes	100%;0%;1
22	In my country, patients (or their caregivers) tend to prefer moderately accessible innovative treatments like Zolgensma—those available within reasonable timeframes and costs—when there is some evidence of effectiveness, even if it is not yet fully proven.	No	40%;10%;2
23	In my country, patients (or their caregivers) tend to prefer less accessible innovative treatments like Zolgensma— wait longer for evidence generation and potentially more costly — but with higher certainty around the evidence.	No	67%;11%;2

*% **Agreement** = Sum of "Agree" + "Strongly Agree" responses; **% Disagreement** = Sum of "Disagree" + "Strongly Disagree" responses; **I Don't Know** = Number of "I don't know" responses

Section S2: Delphi Statements That Reached Consensus

**N**	**Round**	**Section**	**Statements**	*% agreement, % disagreement, I don't know**
1	*R1*	*Population*	The group of patients that receive Zolgensma should include pre-symptomatic patients identified through newborn screening.	91%;9%;0
2	*R1*	*Intervention*	The single, intravenous infusion of Zolgensma should be administered in a specialized clinic or hospital, by a healthcare professional with experience in SMA.	92%;0%;0
3	*R1*	*Intervention*	A pharmacological treatment plan for SMA should be comprehensive of pre-treatment screening to confirm diagnosis, dosing regimen based on patients' characteristics (e.g. age, weight, previous treatment, social context), and best supportive care.	100%;0%;0
4	*R1*	*Intervention*	After the administration of Zolgensma, it's important to have regular check-ups to keep track of patient's health and address any side effects that might happen.	100%;0%;1
5	*R1*	*Intervention*	Support to caregivers of a patient with SMA, such as emotional and educational support, is important to maximise the treatment's benefits for the patient and manage any challenge caused by the treatment and/or the condition.	100%;0%;0
6	*R1*	*Intervention*	Although they help with gene therapy, treatment with corticosteroids can be challenging as they can cause side effects (such as bone weakness, delayed growth, weight gain) that add an additional burden to the patient and their caregiver.	82%;0%;1
7	*R1*	*Intervention*	The additional burden posed by the side effects of corticosteroids, which are often used alongside gene therapy, should be considered in the overall evaluation of gene therapy.	90%;0%;2
8	*R1*	*Comparator*	Best supportive care including physical therapy, respiratory support, and nutritional support should be assessed in addition to any relevant comparator for SMA patients.	92%;8%;0
9	*R1*	*Comparator*	No treatment, including historical control groups or untreated cases, could also be considered the appropriate comparator for SMA patients.	90%;10%;2
10	*R1*	*Outcomes*	Primary outcomes should include improvement in motor function (e.g., CHOP-INTEND score, Hammersmith Functional Motor Scale, RULM score, ability to stand and walk).	92%;0%;0
11	*R1*	*Outcomes*	Primary outcomes should include survival without permanent ventilation (particularly for SMA Type 1).	100%;0%;1
12	*R1*	*Outcomes*	Secondary outcomes should include respiratory function and independence from respiratory support.	100%;0%;0
13	*R1*	*Outcomes*	Secondary outcomes should include quality of life improvements for patients such as physical abilities (incl. pain and fatigue), emotional well-being, social participation, and overall daily living activities.	92%;0%;0
14	*R1*	*Outcomes*	Secondary outcomes should include adverse events and safety profile.	100%;0%;0

15	*R1*	*Study Designs*	Randomized controlled trials (RCTs), comparing Zolgensma to another treatment, are the gold standard for assessing the efficacy and safety of Zolgensma.	83%;17%;0
16	*R1*	*Study Designs*	Real-world evidence (RWE) studies are essential for capturing the long-term effectiveness and safety of Zolgensma in everyday clinical practice and should be an important part of the evidence package.	100%;0%;0
17	*R1*	*Study Designs*	Real-world evidence (RWE) studies should involve patients across a broad range of EU countries, using data from clinical records, patient registries, and other real-world sources.	92%;8%;0
18	*R1*	*Study Designs*	Patient-reported outcomes (PRO) studies are essential for understanding the impact of Zolgensma on quality of life from the patient's and caregiver's perspectives and should be an important part of the evidence package.	100%;0%;0
19	*R1*	*Study Designs*	Patient-reported outcomes (PRO) studies should include patients from a broad range of EU countries and should assess patients inputs and preferences on factors such as physical abilities, emotional well-being, social participation, and daily living activities.	92%;8%;0
20	*R1*	*Study Designs*	Patients are open to incorporate insufficient evidence from traditional study designs (such as RCTs) with innovative research methods (such as Single-arm trials, RWE and PRO research) that might provide useful in providing additional evidence for decision-making.	91%;0%;1
21	*R2*	*Population*	The group of patients that receive Zolgensma should exclusively be decided by specialised medical doctors (i.e. a neurologist) that rely their decision on clinical evidence and in consultation with patients and/or their caregivers.	90%;10%;0
22	*R2*	*Intervention*	The effect of treatment, its safety, formulation, dosing, administration characteristics (e.g. one or multiple shots), the burden of treatment for the patient, patient's comorbidity(ies), are all factors that influence patient preference for the type of intervention we receive.	100%;0%;0
23	*R2*	*Intervention*	Patient support programs and availability of information for patients and their caregivers should be evaluated as part of the intervention characteristics.	100%;0%;0
24	*R2*	*Intervention*	Support to caregivers such as the availability of information regarding different treatment options and financial support are important to maximise the treatment's benefits for the patient and manage any challenge caused by the treatment and/or the condition.	100%;0%;0
25	*R2*	*Intervention*	In my country, patients with SMA have access to gene therapy provided by experienced doctors.	90%;10%;0
26	*R2*	*Comparator*	Patients and their families should be involved in deciding the right comparator for Zolgesma along with specialised medical doctors.	100%;0%;0
27	*R2*	*Outcomes*	Patients' and caregivers' preferences on treatment outcomes such as the treatment impact on patients' Quality of Life, play a vital role in choosing the right comparator.	100%;0%;0
28	*R2*	*Outcomes*	Primary outcomes should include achievement of motor milestones (e.g., sitting unassisted for SMA Type 1, standing and walking for SMA Type 2).	90%;0%;0
29	*R2*	*Outcomes*	Quality of life of the caregivers is an important secondary outcome and should be considered.	90%;0%;0

30	*R2*	*Outcomes*	Patients' and caregivers' priorities differ based on their SMA type and patient's characteristics.	100%;0%;0
31	*R2*	*Outcomes*	There is a need to develop new scales that are able to capture more granular improvements in motor skills due to the lack of sensitivity of current scales to measure small progresses.	100%;0%;0
32	*R2*	*Outcomes*	Patients believe it is important to conduct RCTs within Europe to ensure they have the opportunity to participate in trials across the region, regardless of the specific country where the research is conducted.	89%;0%;0
33	*R2*	*Study Designs*	Patients and caregivers are open to incorporate insufficient evidence from traditional study designs (such as RCTs or Single-arm trials) with innovative research methods (such as RWE and PRO research) that might provide additional evidence for decision-making.	100%;0%;0
34	*R2*	*Study Designs*	New SMA trials, including those for gene therapies, are often limited to treatment-naive patients (patients that did not receive any previous treatment for SMA). This poses a significant issue because most patients are already on SMN-targeted therapies. It would be more beneficial if these trials were designed to also include patients who already had previous treatments.	90%;0%;0
35	*R3*	*Population*	As a minimum standard, Zolgensma should be available to patients that fit the inclusion criteria used in the SMART study; the main trial evaluating Zolgensma use in children who have been diagnosed with SMA, confirmed by a mutation in the SMN1 gene, and weigh up to 21 kg.	
36	*R3*	*Population*	A patient's age alone should not determine their eligibility for Zolgensma treatment.	100%;0%;0
37	*R3*	*Intervention*	Ideally, gene therapy administration for SMA should involve a multidisciplinary team comprised of healthcare professionals experienced in both SMA and gene therapies.	100%;0%;0
38	*R3*	*Intervention*	Access to healthcare centers offering gene therapy varies significantly across European countries. While some countries have sufficient number of centers with good access, other countries face poor access and limited availability of centers.	100%;0%;0
39	*R3*	*Intervention*	In some cases, the treatment and follow-up care for patients undergoing gene therapy impose significant indirect costs on parents and caregivers, primarily due to time away from work which may negatively impact their salaries.	92%;0%;0
40	*R3*	*Comparator*	Patients and caregivers should receive high-quality, harmonized and comprehensive information on the risks and benefits of different treatment options for SMA in my country to make an informed decision.	100%;0%;0
41	*R3*	*Study Designs*	Single-arm trials, where all participants receive the same treatment (Zolgensma), with no comparison group or placebo, are an acceptable method for understanding the treatment's effects from a patient point of view.	100%;0%;0
42	*R3*	*Study Designs*	It is important to conduct single-arm trials within Europe to ensure that Europe-based patients have the opportunity to participate in trials, regardless of the specific country where the research is conducted.	83%;17%;0
43	*R3*	*Study Designs*	Randomised controlled trials (RCTs), comparing Zolgensma to another treatment are the gold standard for assessing the efficacy and safety of Zolgensma.	100%;0%;1

*% **Agreement** = Sum of “Agree” + “Strongly Agree” responses; **% Disagreement** = Sum of “Disagree” + “Strongly Disagree” responses; **I Don’t Know** = Number of “I don’t know” responses

Section S3: Delphi Statements That Did Not Reach Consensus

N	Round	Section	Statements	*% agreement, % disagreement, I don't know**
1	*R1*	*Population*	The group of patients that receive Zolgensma should exclusively be decided by specialised medical doctors that rely their decision on clinical evidence	75%;25%;0
2	*R1*	*Population*	The group of patients that receive Zolgensma should include symptomatic patients, across all SMA types, identified through genetic testing, but the inclusion criteria should focus on age, weight, previous treatment, motor function, and comorbidity.	67%;17%;0
3	*R1*	*Population*	Zolgensma should be available exclusively to patients that fit the inclusion criteria used in the SMART study, the main trial evaluating Zolgensma use in children up to 9 years old who have been diagnosed with SMA, confirmed by a mutation in the SMN1 gene, that weight up until 21 kg and regardless of motor function.	33%;58%;0
4	*R1*	*Intervention*	How a treatment works, its formulation, dosing, side effects, and how it is given are all factors that influence patient preference for the type of intervention we receive.	75%;0%;0
5	*R1*	*Intervention*	A treatment like Zolgensma, which works by fixing the genetic problem causing SMA, is preferrable to a treatment that focuses on treating the symptoms and slowing the progression of the disease.	67%;17%;0
6	*R1*	*Intervention*	The administration of Zolgensma, which requires a single intravenous infusion (IV) at the hospital, is preferred to an oral treatment, taken daily at home, for life.	55%;18%;0
7	*R1*	*Intervention*	The single, intravenous infusion of Zolgensma should be administered in a specialized clinic or hospital, by a healthcare professional with experience in gene therapy.	75%;8%;0
8	*R1*	*Intervention*	SMA patients experience difficulties in accessing gene therapy treatment in my country.	17%;50%;0
9	*R1*	*Intervention*	Getting to the treatment centre for gene therapy can be a big challenge for some SMA patients and their caregivers in my country.	25%;75%;0
10	*R1*	*Intervention*	Accessing gene therapy can be challenging in my country because not all healthcare providers (HCPs) are familiar with it.	33%;58%;0
11	*R1*	*Intervention*	In my country, treatment for SMA carries significant indirect costs, such as reduced working time and transportation, for patients and their families.	75%;25%;0
12	*R1*	*Intervention*	The indirect costs of SMA treatment should be considered in the overall evaluation of gene therapy.	50%;50%;0
13	*R1*	*Comparator*	The right comparator for Zolgensma should exclusively be decided by specialized medical doctors that rely their decision on clinical evidence.	50%;42%;0
14	*R1*	*Comparator*	Nusinersen, administered via intrathecal injection with a loading dose followed by maintenance doses every four months, is the appropriate comparator for SMA patients.	50%;33%;0
15	*R1*	*Comparator*	Risdiplam, administered as a daily oral solution based on weight, is the appropriate comparator for SMA patients.	50%;33%;0

16	*R1*	*Comparator*	Patients receive sufficient information (including potential risks and benefits) about the different treatment options for SMA in my country.	50%;50%;0
17	*R1*	*Outcomes*	Secondary outcomes should include achievement of motor milestones (e.g., sitting unassisted for SMA Type 1, standing and walking for SMA Type 2)	75%;0%;0
18	*R1*	*Outcomes*	Secondary outcomes should include quality of life improvements for caregivers such as emotional well-being, social participation, reduced burden and access to resources.	75%;8%;0
19	*R1*	*Study Design*	Randomized controlled trials (RCTs), comparing Zolgensma to another treatment must be conducted in EU countries.	33%;67%;0
20	*R1*	*Study Design*	Randomized controlled trials (RCTs), comparing Zolgensma to another treatment must allow patients to switch arm during the trial.	78%;0%;3
21	*R1*	*Study Design*	Single-arm trials, where all participants receive Zolgensma, are the best method for understanding the treatment's effects from a patient point of view, but should include children from a broad range of EU countries.	70%;10%;2
22	*R2*	*Population*	The group of patients with SMA that receive Zolgensma should include symptomatic patients, across all SMA types, regardless of age, identified through genetic testing, and meet the inclusion criteria for weight, previous treatment, and motor function.	78%;22%;0
23	*R2*	*Population*	Zolgensma should be available to patients that fit the inclusion criteria used in the SMART study; the main trial evaluating Zolgensma use in children who have been diagnosed with SMA, confirmed by a mutation in the SMN1 gene, and wiegh up to 21 kg.	80%;10%;0
24	*R2*	*Population*	Age should not be a restrictive factor for the selection of patients that can receive Zolgensma, although individual patient preferences should be considered.	60%;30%;0
25	*R2*	*Population*	The selection of patients for Zolgensma treatment should consider the status of their motor and respiratory functions. These functions should not be severely impaired and there should still be the possibility to gain meaningful improvements.	40%;60%;0
26	*R2*	*Intervention*	A treatment like Zolgensma, which aims at delivering a functional copy of the SMN1 gene, improving motor function, and acheiving developmental milestones, is generally preferable to a treatment that works by targeting the SMN2 gene production of SMN protein and aims at treating the symptoms and slowing the progression of the disease.	40%;10%;0
27	*R2*	*Intervention*	The administration of Zolgensma, which requires a single intravenous infusion (IV), is preferred to a treatment that requires multiple spinal injections (typically, 4 injections over the first two months, followed by a maintainace injection every 4 months, for life).	50%;20%;0
28	*R2*	*Intervention*	The administration of Zolgensma, which requires a single intravenous infusion (IV) at the hospital, is preferred to an oral treatment, taken daily at home, for life.	40%;30%;0
29	*R2*	*Intervention*	For the delivery of a gene therapy, it is more important to have healthcare professionals with experience in SMA and IV infusions than healthcare professionals with experience in gene therapy.	70%;20%;0

30	*R2*	*Intervention*	There are sufficient centres offering gene therapy in my country, and they are adequately distributed across the country.	70%;10%;0
31	*R2*	*Intervention*	The treatment and follow-up care for patients undergoing gene therapy impose significant indirect costs on parents and caregivers, primarily due to time away from work which may negatively impact their salaries.	60%;0%;0
32	*R2*	*Intervention*	It is not necessary to assess the indirect costs of SMA gene therapy treatment for patients and caregivers in the evaluation of the therapy.	0%;67%;0
33	*R2*	*Comparator*	Patients and their families should be involved in deciding the right comparator for Zolgesma along with specialised medical doctors.	33%;56%;0
34	*R2*	*Comparator*	Risdiplam, which has a different mechanism from Zolgesma, and is administered as a daily oral solution based on weight, is not a relevant comparator for patients with SMA because they are used across different patient groups and for different purposes.	33%;56%;0
35	*R2*	*Comparator*	Risdiplam, which has a different mechanism from Zolgesma, and is administered as a daily oral solution based on weight, is not a relevant comparator for patients with SMA because they are used across different patient groups and for different purposes.	80%;20%;0
36	*R2*	*Outcomes*	Patients and caregivers in my country generally prioritise overall survival and motor skills improvement, followed by improvement in respiratory abilities, survival without long-term ventilation, physical and emotional quality of life, and the safety profile. Less priority is given to caregiver quality of life and access to support resources	60%;20%;0
37	*R2*	*Study Design*	Ideally, Randomised controlled trials (RCTs), comparing Zolgensma to another treatment are the gold standard for assessing the efficacy and safety of Zolgensma.	60%;10%;0
38	*R2*	*Study Design*	Patients are generally comfortable with data from RCTs, regardless of where they are conducted globally, provided that the highest standards of quality and ethical conduct are upheld.	70%;10%;0
39	*R2*	*Study Design*	Single-arm trials, where all participants receive Zolgensma, are an acceptable method for understanding the treatment's effects from a patient point of view.	70%;10%;0
40	*R2*	*Study Design*	Patients believe it is important to conduct Single-arm trials within Europe to ensure they have the opportunity to participate in trials across the region, regardless of the specific country where the research is conducted.	70%;10%;0
41	*R2*	*Study Design*	In my country, patients (or their caregivers) would prefer moderately accessible innovative treatment such as Zolgensma (available within average timelines and/or average costs) with moderate certainty (some evidence about how well it works, but not fully proven).	38%;25%;0
42	*R2*	*Study Design*	In my country, patients (or their caregivers) would prefer less accessible innovative treatment such as Zolgensma (wait longer for evidence generation and/or more costly) with higher certainty (strong evidence that it works well).	67%;11%;0
43	***R3*****	***Population***	**When selecting patients for Zolgensma treatment, it's important to consider their motor and respiratory functions. While these functions should ideally not be**	67%;8%;0

			**severely impaired, patients with significant respiratory issues should not be automatically excluded, as any improvement can be meaningful for them.**	
44	*R3***	*Intervention*	**A treatment like Zolgensma, which aims at delivering a functional copy of the SMN1 gene, improving motor function, and achieving developmental milestones, is generally preferable to a treatment that works by targeting the SMN2 gene production of SMN protein and aims at treating the symptoms and slowing the progression of the disease. However, the choice of treatment should ultimately be made on an individual basis, taking into account personal preferences and characteristics.**	67%;17%;0
45	*R3***	*Intervention*	**The administration of Zolgensma, which requires a single intravenous infusion (IV), is generally preferred over treatments that require multiple spinal injections (typically, 4 injections over the first two months, followed by a maintenance injection every 4 months for life). However, the choice of treatment should ultimately be made on an individual basis, taking into account personal preferences and characteristics.**	67%;25%;0
46	*R3***	*Intervention*	**The administration of Zolgensma, which requires a single intravenous infusion (IV) at the hospital, is preferred to an oral treatment, taken daily at home, for life. However, the choice of treatment should ultimately be made on an individual basis, taking into account personal preferences and characteristics.**	50%;17%;0
47	*R3***	*Intervention*	**The indirect costs associated with SMA gene therapy should be factored into the overall assessment of the treatment.**	58%;17%;0
48	*R3***	*Intervention*	**For patients receiving SMA gene therapy, including indirect costs in drug assessments is important, whereas caregivers place less emphasis on these costs**	55%;9%;0
49	*R3***	*Comparator*	**Nusinersen which has a different mechanism from Zolgensma, and is administered every four months, is not a relevant comparator for patients with SMA because these products are used across different patient groups and for different purposes.**	33%;50%;0
50	*R3***	*Comparator*	**Risdiplam, which has a different mechanism from Zolgensma, and is administered as a daily oral solution based on weight, is not a relevant comparator for patients with SMA because they are used across different patient groups and for different purposes.**	33%;50%;0
51	*R3***	*Outcomes*	**When evaluating a therapy for SMA, patients and caregivers typically prioritise aspects like overall survival and motor skills improvements over the caregiver's quality of life and access to support resources.**	50%;42%;0
52	*R3***	*Study Design*	**Patients are generally comfortable with data from Randomized Control Trials, regardless of where they are conducted, provided that the highest standards of quality and ethical conduct are upheld.**	58%;25%;0
53	*R3***	*Study Design*	**Patients may choose to stay close to family rather than traveling for the most effective treatment, especially when multiple options are available, even if less effective.**	73%;18%;1

54	*R3***	*Study Design*	**In general, patients (or their caregivers) prefer innovative treatments like Zolgensma, even if they are less accessible due to higher costs or due to the need for more evidence.**	75%;17%;0
55	*R3***	*Study Design*	**In my country, patients (or their caregivers) tend to prefer moderately accessible innovative treatments like Zolgensma—those available within reasonable timeframes and costs—when there is some evidence of effectiveness, even if it is not yet fully proven.**	40%;10%;2
56	*R3***	*Study Design*	**In my country, patients (or their caregivers) tend to prefer less accessible innovative treatments like Zolgensma—wait longer for evidence generation and potentially more costly — but with higher certainty around the evidence.**	67%;11%;2

***% Agreement** = Sum of "Agree" + "Strongly Agree" responses; **% Disagreement** = Sum of "Disagree" + "Strongly Disagree" responses; **I Don't Know** = Number of "I don't know" responses

**Final statements that did not reach consensus after 3 rounds

Section S4: The Role of the Ranking Exercises

As part of the first round of the Delphi process, participants were invited to complete structured ranking exercises to quantitatively assess their preferences regarding (1) the prioritization of outcomes relevant to CAR-T therapy in SMA and (2) their willingness to trade off uncertainty in the evidence base for earlier access to innovative treatments. These exercises were accompanied by semi-structured interviews to qualitatively explore the reasoning behind each participant's responses.

Outcome Prioritization

The ranking of outcomes revealed a strong and consistent prioritization of core clinical endpoints, particularly overall survival, improvements in motor function and respiratory abilities, and survival without permanent ventilation. Outcomes related to quality of life, including both physical and emotional well-being, along with safety profile, were also rated highly, though with greater variability.

Qualitative interviews revealed that these lower rankings did not imply a lack of importance but rather reflected a prioritization of urgent clinical needs. The qualitative analysis also revealed distinct differences between participant profiles, particularly between patients and caregivers. Patient representatives tended to emphasize outcomes related to functional autonomy, social inclusion, caregiver burden and quality of life including daily living activities, reflecting lived experience with progressive disability. Caregivers, on the other hand, often placed greater emphasis on clinical outcomes, emotional well-being, and the need for supportive services, particularly in the context of long-term care.

This divergence is just indicative, and further research is needed. Nonetheless, it highlights the heterogeneity of perspectives within the SMA community, underlining the importance of including both patients and caregivers in HTA processes to help ensure comprehensive representation of stakeholder values.

Trade-offs Between Evidence and Access

Contrary to common assumptions in rare disease contexts, most participants did not express a general willingness to accept lower levels of evidence certainty in exchange for earlier access to CAR-T therapy. While some degree of flexibility was acknowledged, particularly in life-threatening or highly disabling conditions, the prevailing view among panelists favored robust evidence to guide treatment decisions. Several participants emphasized that the landscape of SMA treatments has evolved significantly in recent years, with more approved therapeutic options now available, reducing the perceived urgency to accept immature or incomplete evidence for new interventions.

The results also suggest geographic variability in attitudes toward evidence-access trade-offs, with panelists from countries with slower or more restricted access to advanced therapies expressing slightly more openness to trading certainty for availability. However, these results seem to be just indicative, and further studies and analysis are needed to confirm these findings.

The qualitative interviews further revealed that preferences regarding uncertainty were highly individual and outcome dependent. For instance, some participants indicated that some patients would be more willing to accept uncertainty for therapies targeting survival or motor milestones, but less so for treatments promising incremental or quality-of-life benefits. Similarly, personal risk tolerance, experience with the healthcare system, and perceptions of unmet need were all factors influencing willingness to accept trade-offs.

Implications for Delphi Rounds 2 and 3

The results of the ranking exercises, enriched by interview data, directly informed the refinement and expansion of statements in subsequent Delphi rounds. Statements were reworded to better reflect value-based trade-offs, new themes were introduced (e.g., support programs, caregiver quality of life, indirect costs, trial inclusivity, geographic accessibility), and areas of known divergence were retained for continued discussion. Importantly, early exposure to the diversity of views during Round 1 facilitated greater reflexivity among panelists, enabling a more productive path toward consensus in the later stages of the Delphi process.

Section S5: Qualitative Insights

The qualitative data collected through open-ended responses across the Delphi rounds provided critical context for interpreting the consensus results and illuminated areas of ongoing tension, complexity, and unmet need. These insights enhanced the depth of the findings by capturing subjective and experiential dimensions of treatment preferences that were not fully represented in structured rating scales.

First, many participants emphasized the importance of shared decision-making, calling for clinical frameworks that integrate patient and caregiver perspectives into eligibility decisions for CAR-T therapy. Respondents stressed the need for greater flexibility beyond rigid clinical trial inclusion criteria, noting that real-world patient needs often diverge from standardized protocols. Several comments underscored that eligibility should not be solely based on motor or respiratory function but should also reflect personal values, treatment goals, and family context.

Second, a recurring theme was the emotional and logistical burden of living with SMA—particularly in relation to navigating treatment access, managing uncertainty, and coordinating care across healthcare systems. Participants noted that these burdens vary significantly by country and social support structures, which can influence both treatment outcomes and decision-making processes. These insights reinforced the importance of designing HTA frameworks that reflect equity of access and real-world feasibility, especially for cross-border patient communities in the EU.

Third, patients and caregivers repeatedly highlighted gaps in the availability and quality of information, particularly regarding comparator options and long-term expectations of CAR-T and other therapies. Participants requested clearer, harmonized communication from healthcare providers and manufacturers to support informed choices, especially given the complexity and novelty of gene and cell therapies. This aligns with prior literature on the role of information access in treatment adherence and satisfaction.

Finally, participants criticized the inadequacy of existing outcome measures in capturing meaningful change for patients. Standardized instruments were often seen as too narrow or insensitive, failing to reflect small but significant functional gains, emotional well-being, or improvements in daily participation. Panelists advocated for the development of more holistic, patient-centered metrics that incorporate lived experience and quality of life beyond clinical endpoints.

Section S6: ACCORD Guidelines

Item No.	Section	Checklist Item (*help text*)
T1	**Title**	Identify the article as reporting a consensus exercise and state the consensus methods used in the title.<br>*For example, Delphi or nominal group technique.*
I1	**Introduction**	Explain why a consensus exercise was chosen over other approaches.
I2		State the aim of the consensus exercise, including its intended audience and geographical scope (national, regional, global).
I3		If the consensus exercise is an update of an existing document, state why an update is needed, and provide the citation for the original document.
M1	**Methods**<br>Registration	If the study or study protocol was prospectively registered, state the registration platform and provide a link. If the exercise was not registered, this should be stated.<br>*Recommended to include the date of registration.*
M2	Selection of Steering Committee and/or panellists	Describe the role(s) and areas of expertise or experience of those directing the consensus exercise.<br>*For example, whether the project was led by a chair, co-chairs or a steering committee, and, if so, how they were chosen. List their names if appropriate, and whether there were any subgroups for individual steps in the process.*
M3		Explain the criteria for panellist inclusion and the rationale for panellist numbers. State who was responsible for panellist selection.
M4		Describe the recruitment process (how panellists were invited to participate).<br>*Include communication/advertisement method(s) and locations, numbers of invitations sent, and whether there was centralised oversight of invitations or if panellists were asked/allowed to suggest other members of the panel.*
M5		Describe the role of any members of the public, patients or carers in the different steps of the study.
M6	Preparatory research	Describe how information was obtained prior to generating items or other materials used during the consensus exercise.<br>*This might include a literature review, interviews, surveys, or another process.*
M7		Describe any systematic literature search in detail, including the search strategy and dates of search or the citation if published already.<br>*Provide the details suggested by the reporting guideline PRISMA and the related PRISMA-Search extension.*
M8		Describe how any existing scientific evidence was summarised and if this evidence was provided to the panellists.
M9	Assessing consensus	Describe the methods used and steps taken to gather panellist input and reach consensus (for example, Delphi, RAND-UCLA, nominal group technique).<br>*If modifications were made to the method in its original form, provide a detailed explanation of how the method was adjusted and why this was necessary for the purpose of your consensus-based study.*
M10		Describe how each question or statement was presented and the response options. State whether panellists were able to or required to explain their responses, and whether they could propose new items.<br>*Where possible, present the questionnaire or list of statements as supplementary material.*
M11		State the objective of each consensus step.<br>*A step could be a consensus meeting, a discussion or interview session, or a Delphi round.*
M12		State the definition of consensus (for example, number, percentage, or categorical rating, such as 'agree' or 'strongly agree') and explain the rationale for that definition.
M13		State whether items that met the prespecified definition of consensus were included in any subsequent voting rounds.

M14		For each step, describe how responses were collected, and whether responses were collected in a group setting or individually.
M15		Describe how responses were processed and/or synthesised.<br>*Include qualitative analyses of free-text responses (for example, thematic, content or cluster analysis) and/or quantitative analytical methods, if used.*
M16		Describe any piloting of the study materials and/or survey instruments.<br>*Include how many individuals piloted the study materials, the rationale for the selection of those individuals, any changes made as a result and whether their responses were used in the calculation of the final consensus. If no pilot was conducted, this should be stated.*
M17		If applicable, describe how feedback was provided to panellists at the end of each consensus step or meeting.<br>*State whether feedback was quantitative (for example, approval rates per topic/item) and/or qualitative (for example, comments, or lists of approved items), and whether it was anonymised.*
M18		State whether anonymity was planned in the study design. Explain where and to whom it was applied and what methods were used to guarantee anonymity.
M19		State if the steering committee was involved in the decisions made by the consensus panel.<br>*For example, whether the steering committee or those managing consensus also had voting rights.*
M20	Participation	Describe any incentives used to encourage responses or participation in the consensus process.<br>*For example, were invitations to participate reiterated, or were participants reimbursed for their time.*
M21		Describe any adaptations to make the surveys/meetings more accessible.<br>*For example, the languages in which the surveys/meetings were conducted and whether translations or plain language summaries were available*.
R1	Results	State when the consensus exercise was conducted. List the date of initiation and the time taken to complete each consensus step, analysis, and any extensions or delays in the analysis.
R2		Explain any deviations from the study protocol, and why these were necessary.<br>*For example, addition of panel members during the exercise, number of consensus steps, stopping criteria; report the step(s) in which this occurred.*
R3		For each step, report quantitative (number of panellists, response rate) and qualitative (relevant socio-demographics) data to describe the participating panellists.
R4		Report the final outcome of the consensus process as qualitative (for example, aggregated themes from comments) and/or quantitative (for example, summary statistics, score means, medians and/or ranges) data.
R5		List any items or topics that were modified or removed during the consensus process. Include why and when in the process they were modified or removed.
D1	Discussion	Discuss the methodological strengths and limitations of the consensus exercise.<br>*Include factors that may have impacted the decisions (for example, response rates, representativeness of the panel, potential for feedback during consensus to bias responses, potential impact of any non-anonymised interactions).*
D2		Discuss whether the recommendations are consistent with any pre-existing literature and, if not, propose reasons why this process may have arrived at alternative conclusions.
O1	Other information	List any endorsing organisations involved and their role.
O2		State any potential conflicts of interests, including among those directing the consensus study and panellists. Describe how conflicts of interest were managed.
O3		State any funding received and the role of the funder.<br>*Specify, for example, any funder involvement in the study concept/design, participation in the steering committee, conducting the consensus process, funding of any medical writing support. This could be disclosed in the methods or in the relevant transparency*

		section of the manuscript. Where a funder did not play a role in the process or influence the decisions reached, this should be specified.

Source:

Gattrell WT, Logullo P, van Zuuren EJ, et al. ACCORD (ACcurate COnsensus Reporting Document): A reporting guideline for consensus methods in biomedicine developed via a modified Delphi. *PLoS Med*. 2024;21(1):e1004326. Published 2024 Jan 23. doi:10.1371/journal.pmed.1004326
